# Seedling emergence response of rare arable plants to soil tillage varies by species

**DOI:** 10.1371/journal.pone.0199425

**Published:** 2018-06-25

**Authors:** Joel Torra, Jordi Recasens, Aritz Royo-Esnal

**Affiliations:** Grupo de Malherbologia i Ecologia Vegetal, Dept. HBJ, Agrotecnio, ETSEA, Universitat de Lleida, Lleida, Spain; California State University Fresno, UNITED STATES

## Abstract

Very little information is available on emergence of rare arable plants (RAP) in relation to soil disturbance and seed burial conditions in Europe. This information is essential to design conservation and soil management strategies to prevent the decline of these species in agroecosystems. The objective of this research was to investigate the effect of soil cultivation with burial time on the emergence and seed persistence of RAP. Seeds of 30 RAP species were collected from Spanish arable fields and subjected to two tillage treatments: (a) no soil disturbance, and (b) autumnal soil disturbance down to 10 cm depth every year. The treatments simulated no-till and tilled (disking), respectively. In plots under no-till, RAP seeds were sown at 1-cm depth. In the tilled plots, seeds were sown homogeneously mixed in the top 1–10 cm of soil. The trial was established every two consecutive seasons, and each trial was maintained for two years. Annual cumulative plant emergence was calculated each year; whereas the first trial was monitored for a third year to estimate seed longevity using a persistence index. The response in emergence of the 30 RAP to annual tillage varied among species. With burial time (number of years), higher emergence was observed for seeds sown in tilled soil. This was true across all species, and with strong season effects. The persistence index was correlated with seed weight, species with bigger seeds had low persistence indices while no pattern was observed for small seeded species. Most RAP species, particularly those with high persistence, showed induction of secondary dormancy processes, highlighting the importance of tillage to promote RAP emergence, and hence, seed bank replenishment. Therefore, as time passes the absence of soil tillage may represent a driver of RAP seed bank decline for those species with secondary dormancy processes. In conclusion, it is important to design soil management strategies, such as regular tillage to promote emergence, on a species basis to preserve RAP in Europe.

## Introduction

Declining plant biodiversity in European agroecosystems has often been attributed to agricultural intensification [1]. A particular set of specialist plants characteristically found in arable fields, called segetal species, have suffered the most drastic population decline in recent years, to the point of becoming rare arable plants (RAP) or even locally extinct in many countries [2; 3]. Because characteristically arable species represent key indicators of the natural and aesthetic values of farmland, their occurrence in agro-ecosystems may be an indicator of sustainable land use [1]. Besides having intrinsic value, they also have an important ecological function as a resource for different trophic groups [4]. Nevertheless, identifying which management practices are more beneficial for the conservation of RAP is difficult [5]. The effects of these practices are not fully understood because of RAP rarity, limiting the reliability of the research and statistical analyses [6]. This in turn limits identification of those factors that are most important in RAP decline and conservation.

Increasing use of herbicides is a prominent driver of decline in plant diversity in farming systems [7]. Other contributing factors include shortened rotations to a few highly competitive crops, increased mechanization, improved seed cleaning, landscape simplification, and increased uses of agrochemicals such as fertilizers [8; 9]. Although herbicides may be an important driver of RAP decline, they are not necessarily a direct cause of rarity for all species [10]. Moreover, for other factors such as crop competition and/or fertilizers not all rare segetal species respond in similar ways, which highlights that these factors alone are poor predictors of the rarity of RAP species [11], and others should also be considered.

Among anthropogenic agricultural practices, tillage is one of the main drivers of weed communities [12]. Soil tillage directly affects weed germination and emergence by modifying both the soil properties and the seed distribution in the soil [13]. Moreover, seeds are usually subjected to short light exposures in conjunction with soil cultivation in arable land. Annual weeds can use this short light exposure as a germination cue to detect the optimal time of germination, rather than just proximity to the soil surface [14]. Furthermore, one of the most important means by which weeds survive is through their ability to accumulate seeds and persist in the soil seedbank [15]. Persistence via strong seed dormancy in the soil depends on mechanisms that prevent germination of buried seeds, such as delayed germination, light requirement for germination and reaction to Diurnally Fluctuating Temperatures (DFTs) [16; 17]. Moreover, the proportion of the germinating seed bank depends, among many factors, on the dormancy level of the seed population, which can also be affected by soil disturbance which acts as a termination cue for dormancy release [18]. For example, in a study the arable weed species *Polygonum aviculare* almost disappeared under no-tillage after 22 years while it was very abundant in tilled systems [19]. Thus, tillage system is important for the replenishment of seed banks in species that need light for germination, such as *P*. *aviculare* [20].

Arable flora, such as RAP, have adapted to regularly recurring soil disturbances for thousands of years [21]. With the appearance of herbicides, conservation tillage techniques such as no-till frequently replaced inversion tillage (i.e. ploughing) [22], particularly in semi-arid cereal fields in Mediterranean Europe [23]. However, reduced cultivation and the abandonment of regular tillage in arable fields could have increased the establishment of certain weeds, such as perennials or winter annuals, at the expense of the highly specialized RAP [21]. Compared to no-till, tillage allows incorporation of seeds into the soil seed bank at a depth where seeds are no longer exposed to seasonally varying germination stimuli. Therefore, regular tillage might be crucial to promote emergence and build-up RAP seed reserves, as suggested by some studies [24]. However, almost no information is available regarding the mid to long-term effects of tillage systems on RAP. In Spain, Hernandez-Plaza et al. [25] found that after 23 years *Agrostemma githago*, *Hypecoum procumbens*, and *Roemeria hybrida*, were more abundant or only present in tilled systems, though the number of RAP species or their abundances were too low to understand the effects on this particular set of arable plants. Evidence from the UK suggests tilled agricultural margins with low input and low cover may promote RAP diversity [26]. Thus, how recent changes in soil management (e.g., no-till or the abandonment of mouldboard ploughing) affect RAP is poorly understood.

As a functional group, RAP share a ‘rare weed trait syndrome’ of short-stature, large seeds and late flowering [27]. This concept explains their decline in different parts of Europe based on their similar response to management factors [28]. However, some studies highlight that the rarity of these species can be the result of specific traits of each species [27; 28]. Moreover, while the response of RAP to random factors, such as weather conditions, can explain why they remain in some fields, it can hinder understanding the role of single management strategies on the abundance of each species. Therefore, the response of RAP to different relevant management factors, such as soil disturbance, should be studied at the species level. Studying this species-specific response would provide invaluable information for understanding their rarity and for conservation purposes [21].

In this research, the main objective was to study the effect of simulated soil tillage or no-till on total annual emergence of 30 RAP. The experiment was repeated for two seasons to address temporal variation in emergence due to year-to-year climate differences. One trial was monitored for an additional third year, to understand how short-term seed dormancy and persistence vary across the 30 RAP species studied with burial time.

## Materials and methods

### Plant material

Seeds of 30 RAP species were harvested at maturity from arable fields in Spain from late June through August for two consecutive years (2012 and 2013). Collection sites were located in two areas that host high arable plant diversity: the provinces of Teruel and Lleida. These two areas had been surveyed during 2007–2011, so flowering and seed set dates were well established for all species. The same populations were sampled every year when possible. To ensure randomization, seeds were collected from at least 20 plants throughout the field and mixed in paper bags. The 30 species studied, belonging to eight different botanical families, are listed in Table 1 specifying province of origin, location, and collection dates from each year, while locality coordinates are found in S1 Table. All species studied are rare in the region [2; 5; 6; 8; 10; 18; 29].

**Table 1:** Collection details (province, locality and dates) for 30 rare arable species in Spain for two consecutive years (2012/13 and 2013/14). Number represents the amount of seeds sown per elementary plot each season. Number: amount of seeds sown in each individual plot (0.5 m^2^) every season.

Species	Family	2012/13				2013/14			
		Province	Locality	Date	Number	Province	Locality	Date	Number
*Adonis aestivalis*	Ranunculaceae	Teruel	Camarillas	13/07	200	Teruel	Camarillas	06/08	300
*Adonis flammea*	Ranunculaceae	Lleida	Àger	26/06	200	Teruel	Alpeñés	04/07	300
*Agrostemma githago*	Caryophyllaceae	Teruel	Puerto de Bañón	23/07	500	Teruel	Camarillas	06/08	500
*Androsace maxima*	Primulaceae	Teruel	Camarillas	27/06	1000	Teruel	Hinojosa de Jarque	15/07	500
*Asperula arvensis*	Rubiaceae	Teruel	Puerto de Bañón	10/07	400	Teruel	Camarillas	06/08	400
*Bifora radians*	Apiaceae	Teruel	La Cañadilla	10/07	1000	Teruel	La Cañadilla	06/08	500
*Biscutella auriculata*	Brassicaceae	Teruel	Fuentes Calientes	27/06	200	Teruel	Fuentes Calientes	13/07	200
*Bupleurum rotundifolium*	Apiaceae	Teruel	Puerto de Bañón	10/07	1000	Teruel	Camarillas	06/08	500
*Camelina microcarpa*	Brassicaceae	Teruel	Fuentes Calientes	27/06	1000	Teruel	Camarillas	06/08	500
*Cerastium perfoliatum*	Caryophyllaceae	Teruel	Puerto de Bañón	12/06	1000	Teruel	Fuentes Calientes	04/07	500
*Conringia orientalis*	Brassicaceae	Teruel	Camarillas	27/06	1000	Teruel	Fuentes Calientes	15/07	500
*Consolida orientalis*	Ranunculaceae	Teruel	Camarillas	27/06	1000	Teruel	Camarillas	06/08	500
*Consolida pubescens*	Ranunculaceae	Lleida	Àger	26/06	200	Lleida	Àger	26/06	200
*Delphinium gracile*	Ranunculaceae	Lleida	Bellmunt	06/08	100	Lleida	Alcanó	28/08	200
*Delphinium halteratum*	Ranunculaceae	Lleida	Àger	23/08	500	Lleida	Àger	17/09	300
*Galeopsis ladanum*	Lamiaceae	Teruel	Camarillas	13/07	300	Teruel	La Cañadilla	06/08	200
*Hypecoum pendulum*	Papaveraceae	Teruel	Galve	10/07	200	Teruel	Galve	15/07	200
*Iberis amara*	Brassicaceae	Teruel	Fuentes Calientes	13/07	300	Teruel	Fuentes Calientes	13/07	300
*Lathyrus aphaca*	Fabaceae	Teruel	La Cañadilla	13/08	100	Teruel	La Cañadilla	06/08	100
*Legousia hybrida*	Primulaceae	Lleida	Agulló	29/06	500	Lleida	Agulló	29/06	100
*Neslia paniculata*	Brassicaceae	Teruel	Fuentes Calientes	27/06	1000	Teruel	Fuentes Calientes	15/07	500
*Nigella gallica*	Ranunculaceae	Lleida	Àger	03/10	1000	Lleida	Àger	14/09	300
*Papaver argemone*	Papaveraceae	Teruel	Puerto de Bañón	10/07	1000	Teruel	Fuentes Calientes	04/07	500
*Papaver dubium*	Papaveraceae	Teruel	Puerto de Bañón	10/07	1000	Teruel	Aliaga	04/07	500
*Ranunculus arvensis*	Ranunculaceae	Teruel	Camarillas	10/07	500	Teruel	Camarillas	15/07	400
*Roemeria hybrida*	Papaveraceae	Teruel	Galve	10/07	1000	Teruel	Fuentes Calientes	15/07	500
*Silene conoidea*	Caryophyllaceae	Teruel	Hinojosa de Jarque	27/06	300	Teruel	Camarillas	15/07	500
*Thlaspi arvense*	Brassicaceae	Teruel	Mezquita de Jarque	12/06	1000	Teruel	Fuentes Calientes	15/07	500
*Turgenia latifolia*	Apiaceae	Teruel	Fuentes Calientes	10/07	500	Teruel	Camarillas	06/08	500
*Vaccaria hispanica*	Caryophyllaceae	Teruel	Camarillas	10/07	1000	Teruel	Camarillas	06/08	500

After collection, seeds were air-dried for one week and then stored in dry conditions with silica gel at room temperature (approximately 21°C) until experiments started. After cleaning the seeds selected for the trials, they were counted either manually or with a seed counter (Contador E 230 V; Pfeuffer GmbH, Kitzingen, Germany). The mean weight of 1,000 seeds was estimated for each species by weighing four lots of 100 seeds from the 2012 collections. Seeds visibly inadequate were excluded based on seed firmness (determined by squeezing them with forceps) and the presence of mould [30]; thus, seemingly high-quality seeds were used.

### Experimental design

The collected seeds were sown in a fallow part of a winter cereal field with permission of the owner, with a soil texture determined as 47.4% sand, 18.4% clay, and 34.2% silt, pH 8.2 and 2.9% of organic matter, located in Almenar (Lleida, Spain; 41°46'36" N, and 00°32'07" E). The experiment was replicated in two consecutive seasons, the first initiated on 28 August 2012 (season 2012/13 or S1), and the second on 28 August 2013 (season 2013/14 or S2). This sowing date allowed seeds to experience natural temperature and humidity fluctuations for about two months until typical usual cereal sowing dates in October/November, which corresponds to the beginning/start of the autumnal rainfall period.

RAP seeds were sown according to two experimental tillage treatments: non-till treatment, with seeds sown exactly at 1 cm soil depth, and till treatment, with seeds sown and homogeneously distributed between 1–10 cm soil depths. The 1-cm depth simulated no-till conditions where seeds are maintained at or near the soil surface covering them with 1 cm soil; the contrasting treatment simulated soil tillage (e.g., disking) where seeds are evenly mixed between the 1 to 10 cm soil depths using hoes, the normal depth for minimum tillage in the area [31; 32]. The number of seeds sown in each individual plot varied according to availability among species, but ranged from 100 to 1000 seeds (Table 1).

The treatments were arranged in a completely randomized block design with four replications, in both seasons of the study (S1 and S2). Each RAP species was sown in single plots without crop: four plots in no-till conditions and four plots with soil disturbance. Therefore, each year 240 plots were established and sown with individual species per tillage treatment. Plot dimension was 0.5 by 0.5 m, buffered with 0.5 m corridors between them, and alleys of 1 m width between blocks. On 2 November 2012, 28 October 2013 and 16 October 2014, tilled plots were hoed with hand-tools to simulate a tillage operation, which are usual timings for tillage and sowing operations in the area. Emergence was monitored weekly via destructive counts from the time of soil disturbance until May when emergence ceased. S1 (started in 2012) was monitored over three consecutive years, 2012–15, while S2 (started in 2013) was monitored over two years, 2013–15. Plots were left as fallow after May until the next year.

### Data

Four variables were used to build three types of data sets to be analyzed: (1) first year cumulative emergence after burial was calculated as percentage of sown seeds for S1 in season 2012/2013, and for S2 in season 2013/2014; (2) second year cumulative emergence after one year of burial was calculated for S1 for 2013/14, and for S2 in 2014/15, subtracting previous cumulative emergence the first year; (3) total cumulative emergence over two years after burial was calculated for S1 between 2012–2013 and 2013–2014, and for S2 between 2013–2014 and 2014–2015; and (3) third year cumulative emergence after two years of burial was calculated for S1 for 2014/2015, subtracting previous cumulative emergence from initial sown seeds. Daily precipitation and temperature were obtained from a meteorological station situated 4 km away from the experimental site.

### Statistical analysis

For each species, quasi-binomial Generalized Linear Models (GLMs) using the logit link function were used to test the effect of tillage and season (repetition in two consecutive seasons) treatments on the proportion of cumulative emergence. This procedure allowed the correction of over dispersion in the data. Tillage treatment (no-till and till conditions) and season treatment (S1 and S2) were set as categorical fixed factors and repetitions as a random factor. Analysis was done separately for four sets of data described previously and for the 30 species: the emergence the first year, the emergence the second year, the total emergence after two years, and the total emergence after three years. In the third data set (third year emergence after two years of burial), only tillage treatment was considered as a fixed factor and block as random factor. Seasons are presented and analyzed separately when significant interactions between treatments were found. Statistical analyses were performed in the R environment (R Core Team, 2015) using the package *lmtest* [33].

A regression analysis was performed between the third-year percentage of emergence (PE3) and the weight of 1000 seeds as a proxy for seed size. The aim was to assess if emergence data from seeds already buried two years can be used as an estimator of seed bank persistence for RAP. The percentage of emergence was transformed by means of √(PE3*(number of sown seeds per plot/1000)), called the Persistence Index, and seed weights, as √(1000 seed-weights + 1). The Persistence Index is similar to the Longevity Index defined in previous studies [34], with values close to 0 meaning very low persistence, and higher values indicating high seed persistence. The best fit (R-square and residual plots) was achieved with the following logarithm equation, y = y0 + a ln (x), transforming the data, and using the percentage of emergence with non-till rather than tilled treatment, or averaging both treatments. Sigmaplot 11.0 software was used for regression analysis.

## Results

The three years differed in terms of temperature and precipitation (Table 2). In 2012/13, mean air temperatures were much higher from autumn to spring (at least two-fold) compared to the two following years. The coldest autumn was in 2013/14, while the coldest winter was in 2014/15. In contrast, the wettest autumn was in 2014/15, while the driest was 2013/14. Winter was wettest in 2013/14; while spring was very dry in 2014/15 (Table 2).

**Table 2:** Average temperature and cumulative rainfall for autumn (1st September to 31st December), winter (1st January to 31st March), and spring (1st to 30th April) during three years.

Year	Mean Temperature (°C)			Precipitation (mm)		
	Autumn	Winter	Spring	Autumn	Winter	Spring
2012/13	12.7	6.6	11.9	178	106	78
2013/14	4.0	1.9	5.0	79	171	46
2014/15	5.9	1.1	3.4	297	100	8

Overall, 18 out of the 30 species showed low first year emergence percentages (below 25%), irrespective of tillage and season treatments. Moreover, of those, 8 species had very low emergence rates, below 10%, because under this threshold is considered that most seeds are dormant [35]. Only *Asperula arvensis* showed emergences always greater than 25%. The effect of soil disturbance or tillage (hence seed burial depth), and season on the total emergence the first year differed among species (Table 3). The tillage treatment alone was significant only for *Conringia orientalis* (higher with no-till); the season treatment alone was significant in five species; and the interaction of both treatments alone was significant in three species. Of those, though season was significant, emergence was higher in till plots both years for *Adonis aestivalis* and *Lathyrus aphaca*, and in non-till plots in *Camelina microcarpa* and *R*. *hybrida*. Tillage and the interaction with season were significant in three species, and season and the interaction with tillage only in *A*. *githago*. Finally, significant interactions (tillage with season) were found in 11 species in total; when analyzing the tillage treatment within each season separately, emergence was higher the first one in *Biscutella auriculata* and *Cerastium perfoliatum*, and the second season in five species, while it was higher in tilled plots the first season in another five species (Table 3).

**Table 3:** Total annual cumulative emergence (% ± SE) during the first year after burial of 30 rare arable plants from seeds sown in tilled and no-till plots, the tillage treatment; trials repeated for two consecutive seasons (S1, 2012/13; S2, 2013/14), the season treatment. Soil disturbance was performed in early autumn in tilled plots. The 2^nd^ through 4^th^ columns represent significance for main effects and interactions on emergence analysed by GLM. Values followed by the letter a were significantly higher after analysing each season separately for tillage treatment due to significant interactions.

Species	Tillage	Season	Tillage * Season	Total Cumulative Emergence			
				No-Till		Tilled	
				2012/13	2013/14	2012/13	2013/14
*Adonis aestivalis*	***	**	NS	3.5 ± 0.8	2.1 ± 0.8	14.1 ± 4.5	5.4 ± 2.7
*Adonis flammea*	NS	NS	NS	5.6 ± 1.8	6.9 ± 1.4	2.8 ± 0.6	6.0 ± 3.0
*Agrostemma githago*	NS	***	***	10.0 ± 1.9	52.6 ± 3.0	30.3 ± 4.0a	44.6 ± 5.3
*Androsace maxima*	*	NS	*	9.0 ± 2.1	9.0 ± 2.0a	8.1 ± 1.2	3.3 ± 0.8
*Asperula arvensis*	NS	***	NS	25.2 ± 10.4	55.3± 7.3	35.4 ± 5.0	64.6 ± 1.7
*Bifora radians*	**	***	*	6.0 ± 2.1	31.3 ± 8.3	23.3 ± 3.8a	39.5 ± 5.9
*Biscutella auriculata*	NS	NS	***	2.3 ± 1.3a	6.3 ± 1.2	6.4 ± 1.0a	2.0 ± 0.6
*Bupleurum rotundifolium*	***	***	**	4.3 ± 0.3	41.5 ± 5.4a	5.5 ± 1.4	16.4 ± 3.5
*Camelina microcarpa*	***	**	NS	10.5 ± 4.0	20.3 ± 2.6a	4.0 ± 1.3	7.6 ± 2.3
*Cerastium perfoliatum*	NS	NS	*	5.0 ± 1.0a	1.9 ± 0.5	2.5 ± 0.6	3.0 ± 1.1
*Conringia orientalis*	***	NS	NS	7.9 ± 1.9	11.7 ± 2.0	5.7 ± 1.8	4.1 ± 0.4
*Consolida orientalis*	**	***	NS	9.5 ± 3.1	37.3 ± 9.5	5.9 ± 0.6	16.0 ± 2.3
*Consolida pubescens*	**	**	NS	18.0 ± 6.2	39.5 ± 4.0	15.8 ± 2.8	18.8 ± 3.7
*Delphinium gracile*	NS	***	NS	29.0 ± 8.8	6.1 ± 0.8	16.8 ± 3.3	4.1 ± 1.0
*Delphinium halteratum*	**	*	***	14.7 ± 2.5	26.5 ± 3.7a	13.1 ± 0.6	9.2 ± 1.5
*Galeopsis ladanum*	NS	***	NS	9.2 ± 4.0	3.3 ± 1,1	10.4 ± 1.2	2.6 ± 1.1
*Hypecoum pendulum*	**	***	***	10.1 ± 3.2	13.5 ± 2.0	32.4 ± 4.5a	8.8 ± 2.3
*Iberis amara*	**	***	NS	4.6 ± 1.7	1.1 ± 0.8	10.8 ± 1.3	0.8 ± 0.4
*Lathyrus aphaca*	*	***	NS	35.0 ± 16.5	7.8 ± 5.2	67.0 ± 11.3	21.3 ± 0.6
*Legousia hybrida*	NS	*	NS	3.2 ± 1.1	1.0 ± 0.7	1.8 ± 0.4	1.0 ± 0.5
*Neslia paniculata*	*	***	NS	6.9 ± 4.6	20.9 ± 1.1	5.1 ± 1.4	9.6 ± 0.9
*Nigella gallica*	*	***	NS	24.7 ± 5.9	14.3 ± 3.2	19.1 ± 1.1	7.5 ± 1.0
*Papaver argemone*	***	NS	*	9.8 ± 3.9	14.9 ± 2.0a	6.0 ± 1.1	3.0 ± 0.4
*Papaver dubium*	**	***	NS	4.7 ± 1.6	0 ± 0	1.9 ± 0.7	0 ± 0
*Ranunculus arvensis*	NS	NS	*	11.0 ± 4.1	24.5 ± 6.3	17.0 ± 0.8	14.3 ± 4.5
*Roemeria hybrida*	***	**	NS	10.8 ± 3.3	20.6 ± 3.2	3.6 ± 0.4	2.6 ± 1.0
*Silene conoidea*	NS	NS	NS	3.9 ± 1.4	4.3 ± 0.6	2.8 ± 0.7	3.2 ± 0.4
*Thlaspi arvense*	NS	NS	NS	3.0 ± 0.5	2.9 ± 0.4	2.3 ± 0.6	1.7 ± 0.6
*Turgenia latifolia*	**	NS	***	6.2 ± 1.7	16.6 ± 5.5	26.5 ± 3.7	15.1 ± 2.5
*Vaccaria hispanica*	NS	***	NS	13.1 ± 2.4	42.0 ± 8.0	16.3 ± 1.3a	40.9 ± 3.2

***<0.001,

** 0.001–0.01,

* 0.01–0.05,

NS: not significant.

In contrast, the effect of tillage on total emergence was very different the second year after sowing (S2 Table). Emergences were higher in tilled plots in *Bifora radians*, *C*. *perfoliatum*, *Papaver argemone*, *Ranunculus arvensis* and *Turgenia latifolia*. The season treatment alone was significant in seven species, while the interaction with tillage was significant alone in *Consolida pubescens* and *Delphinium halteratum*. In total, the interaction tillage with season was significant for eight species, with higher emergences in no-till the first season in *Papaver dubium* and *R*. *hybrida*, and the second one in *A*. *aestivalis*, while emergences were higher in tilled plots the first season in *A*. *arvensis* and *C*. *pubescens* and the second one in another four species (S2 Table). Both factors (tillage and season) were significant in six species. Of those, though season was significant, emergence was higher in tilled plots both years for *C*. *orientalis*, and in no-till plots, for *Vaccaria hispanica*. The season and the interaction with tillage were significant in four species. Finally, almost all species (27 out of 30) had emergences below 25%, irrespective of tillage treatment and season, while 17 had very low emergence rates (below 10%).

The total cumulative emergence after two consecutive years of monitoring (Table 4) was significantly higher in tilled soil for *L*. *aphaca*, and in no-till, for *D*. *halteratum*, *P*. *argemone*, and *R*. *hybrida*. The season treatment alone was significant for eight species, and the interaction with tillage alone in three. Overall, the interaction tillage with season was significant in nine species, being emergences higher in no-till only the second season in *Bupleurum rotundifolium* and *C*. *pubescens*, while emergence were higher in tilled plots the first season in five species and the second one in *Thlaspi arvense*. Both factors were significant in 10 species. Of those, though season was significant, emergence was higher in tilled plots both years for *C*. *microcarpa* and *Iberis amara*. After two years, only four species showed very low emergences (<10%) in both season and tillage treatments.

**Table 4:** Total cumulative emergence (% ± SE) after two consecutive years for 30 rare arable plants from seeds buried in no-till and tilled treatments, the tillage treatment; trial repeated in two consecutive seasons (S1, 2012–14; S2, 2013–15), the season treatment. Annual soil disturbance was performed in early autumn in tilled plots. The 2^nd^ through 4^th^ columns represent significance for main effects and interactions with emergence analysed by ANOVA. Values followed by the letter a were significantly higher after analysing each season separately for tillage treatment due to significant interactions.

Species	Tillage	Season	Tillage * Season	Total Cumulative Emergence			
				No-Till		Tilled	
				2012–2014	2013–2015	2012–2014	2013–2015
*Adonis aestivalis*	NS	NS	***	15.3 ± 2.4	42.7 ± 10.1	31.0 ± 3.9a	23.3 ± 5.1
*Adonis flammea*	NS	***	NS	10.0 ± 3.0	22.0 ± 1.8	9.0 ± 2.1	18.7 ± 3.2
*Agrostemma githago*	*	***	***	10.2 ± 1.9	52.8 ± 3.0	30.5 ± 4.1a	44.7 ± 5.4
*Androsace maxima*	NS	NS	NS	11.5 ± 2.7	13.7 ± 2.7	11.0 ± 2.5	6.8 ± 1.2
*Asperula arvensis*	NS	***	NS	25.7 ± 10.5	61.8 ± 7.7	37.4 ± 4.8	67.1 ± 2.4
*Bifora radians*	***	***	NS	6.9 ± 2.2	32.8 ± 7.5	25.5 ± 3.9	42.7 ± 5.4
*Biscutella auriculata*	NS	NS	**	2.6 ± 1.1	7.5 ± 1.8	6.9 ± 1.4a	3.8 ± 1.3
*Bupleurum rotundifolium*	*	***	**	8.3 ± 0.9	46.5 ± 6.7a	11.5 ± 1.6	25.7 ± 4.2
*Camelina microcarpa*	**	**	NS	12.4 ± 4.2	22.7 ± 1.9	6.7 ± 1.2	12.8 ± 3.6
*Cerastium perfoliatum*	NS	NS	*	8.0 ± 2.2	6.7 ± 2.9	5.9 ± 0.9	13.3 ± 2.9
*Conringia orientalis*	NS	*	NS	8.7 ± 1.8	14.4 ± 1.9	7.8 ± 2.2	9.2 ± 0.9
*Consolida orientalis*	*	**	NS	16.7 ± 5.3	39.2 ± 9.7	12.6 ± 1.7	19.4 ± 3.0
*Consolida pubescens*	NS	**	*	19.6 ± 7.3	47.4 ± 4.9a	24.0 ± 4.7	24.5 ± 5.1
*Delphinium gracile*	NS	***	NS	30.5 ± 8.7	7.1 ± 0.8	30.5 ± 12.6	5.5 ± 1.6
*Delphinium halteratum*	*	NS	NS	21.8 ± 4.7	29.7 ± 4.2	18.1 ± 0.5	18.5 ± 3.1
*Galeopsis ladanum*	NS	***	NS	10.9 ± 4.5	4.5 ± 1.9	11.8 ± 1.2	3.3 ± 1.1
*Hypecoum pendulum*	**	***	**	15.8 ± 5.4	14.4 ± 1.8	41.0 ± 6.1a	10.1 ± 2.3
*Iberis amara*	**	***	NS	7.6 ± 1.7	2.8 ± 1.1	14.3 ± 0.5	4.8 ± 1.8
*Lathyrus aphaca*	*	NS	NS	35.0 ± 16.5	55.3 ± 14.0	67.0 ± 11.3	57.0 ± 4.0
*Legousia hybrida*	NS	***	NS	4.2 ± 1.4	32.8 ± 12.4	5.7 ± 1.3	31.3 ± 8.6
*Neslia paniculata*	*	***	NS	9.0 ± 5.2	27.2 ± 2.9	7.6 ± 1.7	15.6 ± 2.2
*Nigella gallica*	NS	***	NS	31.7 ± 5.4	16.4 ± 4.0	23.6 ± 2.7	13.8 ± 1.1
*Papaver argemone*	*	NS	NS	11.9 ± 4.3	15.7 ± 2.0	9.3 ± 1.3	7.4 ± 2.2
*Papaver dubium*	**	***	NS	7.1 ± 1.9	0.2 ± 0.1	2.3 ± 1.0	0.4 ± 0.3
*Ranunculus arvensis*	*	*	NS	16.5 ± 4.5	30.6 ± 7.6	28.3 ± 4.3	32.4 ± 2.2
*Roemeria hybrida*	***	NS	NS	20.1 ± 3.1	21.4 ± 3.0	9.7 ± 1.2	6.6 ± 1.7
*Silene conoidea*	NS	NS	NS	4.9 ± 2.0	6.2 ± 0.6	4.2 ± 0.6	9.0 ± 3.1
*Thlaspi arvense*	**	NS	***	4.6 ± 0.8	3.9 ± 0.5	4.7 ± 1.0	9.7 ± 2.7a
*Turgenia latifolia*	***	NS	***	6.6 ± 1.8	17.0 ± 5.4	27.2 ± 3.9	15.7 ± 2.6
*Vaccaria hispanica*	NS	***	NS	15.2 ± 2.1	42.1 ± 7.9	20.6 ± 1.5a	41.7 ± 2.8

***<0.001,

** 0.001–0.01,

* 0.01–0.05,

NS: not significant.

**Fig 1:**
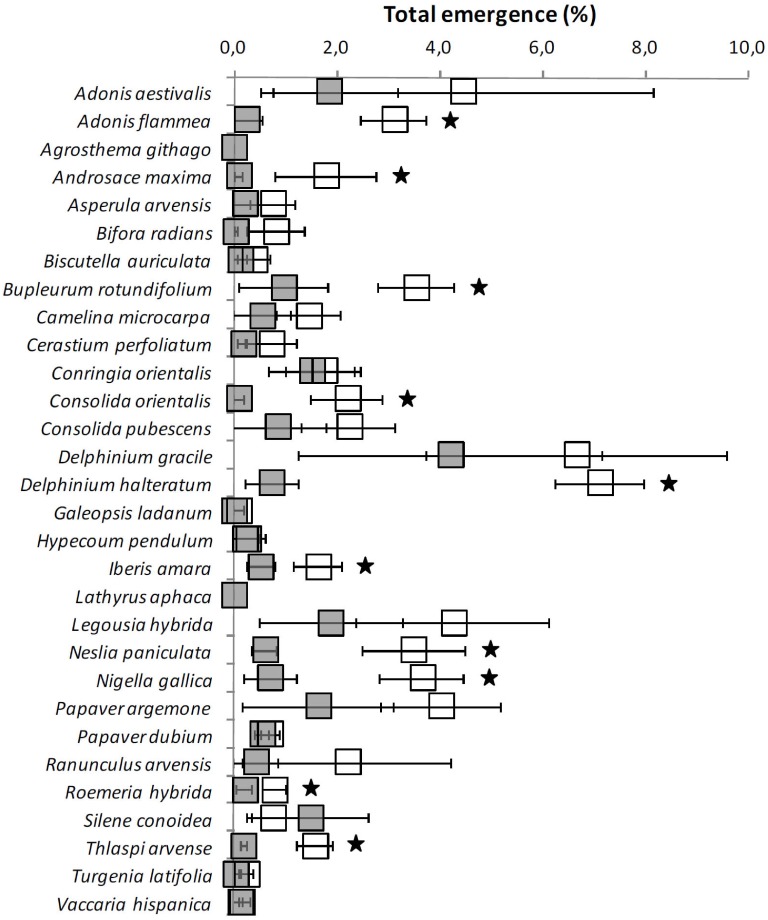
Total cumulative emergence (mean % ± SE) of 30 rare arable plants whose seeds were sown using two tillage treatments (no-till, grey, and tilled once in autumn, white) and emergences monitored a third year 2014/2015 (trial S1); seeds sown at 1–10 cm depth in plots with annual soil disturbance in autumn. Black stars denote significant differences by GLM analyses (P<0.05).

Emergence was only monitored for a third year (2014/15) in the first trial sown in 2012/13, as shown in Fig 1. Twenty species had more emergences with tillage, of which only 10 were statistically significant: *Adonis flammea*, *Androsace maxima*, *B*. *rotundifolium*, *C*. *orientalis*, *D*. *halteratum*, *I*. *amara*, *N*. *paniculata*, *Nigella gallica*, *R*. *hybrida* and *T*. *arvense*.

When comparing the data between years there was a tendency in some species to find higher emergence under no-till the first year while in the following years, particularly the third one, emergence was higher in the tilled treatment. While *B*. *radians*, *C*. *perfoliatum*, *P*. *argemone*, *R*. *arvensis*, *T*. *latifolia* and *V*. *hispanica* did not show differences for the tillage treatment the first year, emergences were higher in tilled plots the second year. For example, *R*. *hydrida* showed higher emergences in no-till the first year while in the third year they were higher in tilled plots.

**Fig 2:**
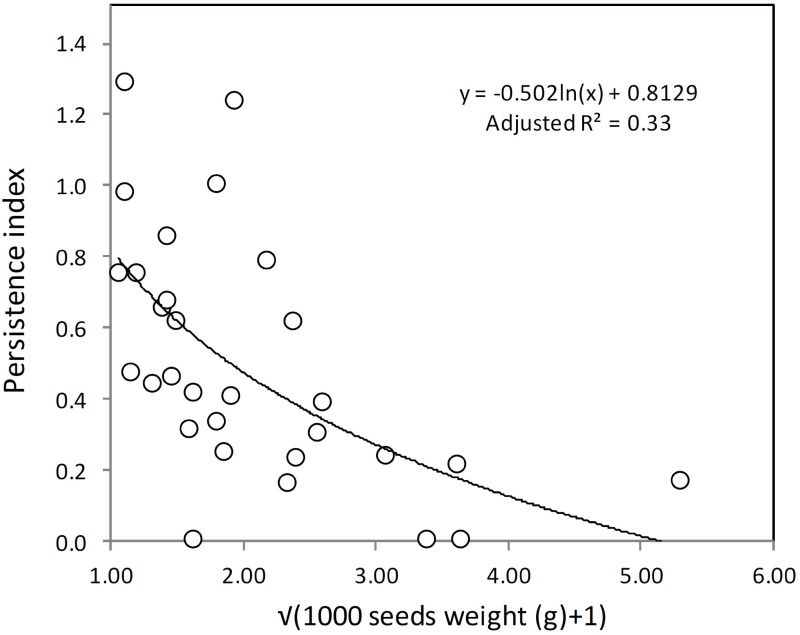
Relationship of the transformed weight of 1000 seeds per species and its persistence index; for details, see text (*F*_1,28_ = 15.4, *P*<0.001). Labels for each point represent the species using the Bayer Code System.

The logarithmic relationship between the transformed weight (g) of 1000 seeds and the transformed persistence index was estimated the year and rendered to be significant (*P*<0.001) and positive (Fig 2), indicating that these two factors are related.

## Discussion

The 30 RAP species studied showed contrasting behaviours without a clear shared pattern in response to tillage in autumn under these study conditions. The effect of the tillage treatment and hence burial depth on total cumulative emergence varied among species. Moreover, for 19 species the responses changed during the second year of burial compared to the first. In the single trial that was monitored a third year (after two years of seed burial) emergence was, in general, greater in simulated tilled soils compared to no-till. A possible explanation for these results is that for a number of RAP species, the light requirement for germination (short light exposure) and seed dormancy levels may change dramatically from one year to the next during burial, depending on the soil disturbance experienced by the seeds in relation to burial time [15].

RAP species can enter into secondary dormancy and may have developed a secondary light requirement for germination with increased burial time and annual tillage [15, 24]. In this study 12 species had higher emergence rates in no-till during the first year (Table 3), only two species (*A*. *arvensis* and *P*. *dubium*) during the second year (S2 Table), and none for the third year (Fig 1). In contrast, six species had significantly more emergence with annual tillage during the first year, and 10 species both in the second and third years. Moreover, looking at the mean emergences the third year, irrespective of the statistical output, they were higher in 23 species in the tilled plots. Light regulates dormancy termination (primary or secondary) and subsequent germination in many weed species [18]. Although many seeds require light for germination at dispersal, light requirement can be generally induced upon burial. It is known that generally cold temperatures during fall and winter induce secondary dormancy in many weed species [24]. A substantial fraction of the 30 RAPs may have displayed this secondary dormancy in this research. This would explain the promotive effect of brief exposure to light of RAP seeds during soil cultivation [18]. The induction of secondary dormancy processes after several months of burial is a common and an important behaviour among RAP [24]. In this study an attempt was made, using the data provided, to classify the 30 RAP species in a qualitative manner according to the presence of secondary dormancy. The criterion used was a balance between average emergences (irrespective of treatments) from the first to the third year (Table 5): species showing at least 50% emergence the second or the third years compared to the first year were classified as having high levels of secondary dormancy; those having at least 25% were considered to have low secondary dormancy; and finally those having less than 25%, as not showing secondary dormancy. As shown in Table 5, 24 species showed secondary dormancy, and of those, 14 showed high degrees of secondary dormancy. In this research, secondary dormancy was mainly detected in the RAP belonging to the Brassicaceae, Papaveraceae or Ranunculaceae families. Secondary dormancy was observed in previous emergence studies for the same *Delphinium* and *Consolida* species and *N*. *gallica* [29], or the same Papaveraceae RAP species [17]. Emergence patterns in other studies with *C*. *microcarpa* [36] or *T*. *arvense* [37] also suggested the presence of secondary dormancy processes.

**Table 5:** Summary of results (% of cumulative emergence, persistence index and weight of 1000 seeds) for 30 rare arable plants during three consecutive years (first and second years of burial [means of seasons S1 and S2]; third year of burial [S1 only]). Percentages represent the yearly emergence irrespective of burial treatment. In the last column species are classified according to the presence of secondary dormancy: no, low or high.

Species	First year	Second year	Third year	Persistence Index	1000 seed weight (g)	Secondary dormancy
*Adonis aestivalis*	6.3	23.0	3.2	0.61	4.6	HIGH
*Adonis flammea*	5.4	10.1	1.7	0.23	4.7	HIGH
*Agrostemma githago*	34.4	0.3	0.0	0	10.4	NO
*Androsace maxima*	7.4	3.7	1.0	0.33	2.2	LOW
*Asperula arvensis*	45.1	6.8	0.5	0.30	5.5	LOW
*Bifora radians*	25.0	2.75	0.4	0.24	8.4	NO
*Biscutella auriculata*	4.2	1.1	0.3	0.16	4.4	LOW
*Bupleurum rotundifolium*	16.9	7.7	2.3	1.00	2.2	HIGH
*Camelina microcarpa*	10.6	3.3	1.0	0.75	0.4	LOW
*Cerastium perfoliatum*	3.1	5.6	0.5	0.44	0.7	HIGH
*Conringia orientalis*	7.3	2.9	1.6	1.23	2.7	LOW
*Consolida orientalis*	17.2	5.7	1.2	0.31	1.5	LOW
*Consolida pubescens*	23.0	8.2	1.5	0.41	1.6	LOW
*Delphinium gracile*	14	5.3	5.5	0.65	0.9	HIGH
*Delphinium halteratum*	15.9	7.2	3.9	0.61	1.2	HIGH
*Galeopsis ladanum*	6.4	1.4	0.0	0	1.6	NO
*Hypecoum pendulum*	16.2	5.5	0.3	0.25	2.4	LOW
*Iberis amara*	4.3	3.3	1.1	0.40	2.6	HIGH
*Lathyrus aphaca*	32.8	24.5	0.0	0	12.2	LOW
*Legousia hybrida*	1.8	16.9	3.1	0.98	0.2	HIGH
*Neslia paniculata*	10.6	4.9	2.1	0.78	3.7	HIGH
*Nigella gallica*	16.4	6.1	2.2	0.85	1.0	HIGH
*Papaver argemone*	8.4	2.9	2.9	1.29	0.2	HIGH
*Papaver dubium*	3.3	0.9	0.6	0.75	0.1	LOW
*Ranunculus arvensis*	14.8	8.2	1.3	0.21	12.0	LOW
*Roemeria hybrida*	9.4	5.4	0.5	0.47	0.3	HIGH
*Silene conoidea*	3.3	2.6	1.1	0.67	1.0	HIGH
*Thlaspi arvense*	2.6	3.3	0.9	0.46	1.1	HIGH
*Turgenia latifolia*	16.1	0.7	0.2	0.17	27.0	NO
*Vaccaria hispanica*	28.1	2.2	0.2	0.39	5.7	NO

Although the presence of secondary dormancy processes could be possible, there is insufficient information in the paper to verify if this was true. The fact that emergence was higher for the second and third year with tillage may be alternatively explained by other factors, as diminishing seed viability [32] or a higher rate of predation for seeds in no-till [38; 39]. The fact that the fate of non-germinated seeds in the soil was not assessed made it difficult to verify the reason/s for the difference observed in emergence between treatments. Moreover, initial dormancy status was unknown and we could not directly evaluate if seeds entered secondary dormancy. Another factor to consider is that experiments were repeated in time (seasons S1 and S2). Seeds were collected every year; therefore, different maturation conditions each year could have contributed to different dormancy status and to differences observed between treatments. Finally, we do not know if seed age changed their secondary light requirement (secondary dormancy) irrespective of burial conditions.

Seed mass has traditionally been related to seed persistence [34]. Based on the positive relationship between the weight of 1000 seeds and emergence during the third year without soil disturbance, the Persistence Index was a rather accurate proxy of seed bank persistence. Weight is an important variable, as is seed size. There may be a confounded effect of size and weight that was not considered in the analysis presented. On the other hand, for the species studied, small seeds were always lighter. In general, persistent seeds tend to be smaller, more compact, dormant and require light for germination, while transient seeds are larger and less dependent on light for germination [34]. The lowest Persistence Index was for species with the larger seeds: *A*. *githago*, *B*. *radians*, *L*. *aphaca*, *R*. *arvensis*, *T*. *latifolia* or *V*. *hispanica* (Table 5). Moreover, none of these species showed greater emergence in tillage treatment the third year. On the other hand, small seeded species (1000 seeds < 2 g) showed a large range of variation for the Persistence Index, and there was a trend for emergence to be tillage dependant with burial time. In this study, those species classified as showing secondary dormancy, particularly high levels, also showed high Persistence Indexes. Therefore, the ability to form persistence seed banks could be related to presence of secondary dormancy processes, to allow seeds to remain dormant in the soil [15, 24].

In this study, trials were replicated in time twice in two consecutive seasons (season treatment). The effect of season treatment on the emergences observed was significant (and with interactions with the tillage treatment) in an important number of the 30 RAP analyzed (the first year, the second or considering both) without a clear pattern. For example, some species showed greater emergence in tilled plots in one season (S1 or S2) without differences between the tillage systems in the other one. Therefore, the response to the tillage treatment was different depending on which season the experiment started (S1 or S2). These results reveal the complexity of interactions between soil factors affected by tillage, such as light signal, and other environmental factors, such as weather, that control dormancy release and seed germination under natural conditions [40]. For example, it is known that the opening of topsoil layers enhances evaporation from the tilled soils and rapid loss of soil water content under high solar radiation [41].

### Implications for management conservation

The effect of soil disturbance changed markedly with burial time across RAP species. Additionally, for some species there is evidence that higher seed mass was related to lower emergence with soil disturbance over burial time, and low soil seed persistence, under these study conditions. This was evident for those species belonging to Cariophyllaceae or Apiaceae families. Soil seed persistence is related to local population extinction, contributing to the vulnerability of species with changing land use [24]. Importantly, emergence was < 10% for 15 species during the first year of burial, with a very steep decline over time; while 26 species showed < 10% emergence during the second year, and no species exceeded this percentage during the third year. Moreover, soil disturbance could be an important driver contributing to the decline of the majority of RAP. This could occur because with burial time, emergence is reduced due to RAP seeds entering into secondary dormancy. Soil tillage could be an important stimulus to promote germination in those seeds, as they are adapted to regular soil disturbances [21]. If seeds remain buried in the soil without germinating for too long, they will finally perish, and, thus, seed banks will be diminished or exhausted. Therefore, the lack of soil tillage could hinder the formation or replenishment of persistent seed banks in RAP [24].

It is important to put these results in perspective, because there are more relevant factors to explain the decline and extinction of RAP, such as the escalating use of herbicides or competition with more aggressive weed species better adapted to modern agriculture [7, 11]. This study highlights the importance of considering soil tillage management as a driver to partially explain changes in RAP abundances. Studies are needed to reveal how the timing, frequency and type of tillage can affect RAP seed banks, or how tillage and environmental factors modulate dormancy processes, considering, for example, that high nitrogen levels can promote protracted emergence [42] and could enhance the depletion of seed banks. From this research it can be concluded that RAP can show contrasting behaviours without a clear shared pattern in response to tillage treatment. For this reason, the effects of soil tillage management should be studied and ascribed on a species basis to improve management and conservation of RAP in Europe.

## Supporting information

S1 Data Data files used in this study. This EXCEL file contains all data used for this manuscript.
(XLSX)

S1 Table Collection localities. Coordinates (North and East) for collection localities of 30 rare arable plant species in Spain.
(DOCX)

S2 Table Proportion of cumulative emergence the second year for 30 rare arable plants. Total annual cumulative emergence the second year (% ± SE) of 30 rare arable plants from seeds sown in till and non-till plots, the tillage treatment; trial repeated in two consecutive seasons (S1, 2013/14; S2, 2014/15), the season treatment. Annual soil disturbance was performed in early autumn in tilled plots sown. The 2nd and 4th columns represent significance for main effects and interactions on emergence analysed by GLM. Values followed by the letter a were significantly higher after analysing each season separately for tillage treatment due to significant interactions.
(DOCX)
