# Supplementary material for: Seedling emergence response of rare arable plants to soil tillage varies by species
Source: PLoS One. 2018 Jun 25;13(6):e0199425. doi: 10.1371/journal.pone.0199425 (PMC6016903; doi:10.1371/journal.pone.0199425)
Supplement: S1 Table — Coordinates (North and East) for collection localities of 30 rare arable plant species in Spain. (DOCX) [file pone.0199425.s002.docx]

|  |  | **Coordinates** | |
| --- | --- | --- | --- |
| **Province** | **Locality** | **North** | **East** |
| Teruel | Camarillas | 40º 37' | 00º 45' |
| Lleida | Alpeñés | 40º 48' | 01º 4' |
| Teruel | Puerto de Bañón | 40º 50' | 01º 9' |
| Teruel | La Cañadilla | 40º 44' | 00º 37' |
| Teruel | Fuentes Calientes | 40º 41' | 00º 57' |
| Lleida | Àger | 42º 00' | 00º 44' / 45' |
| Lleida | Bellmunt | 41º 46' | 00º 58' |
| Teruel | Galve | 40º 41' | 00º 53' |
| Lleida | Agulló | 42º 00' | 00º 43' |
| Teruel | Aliaga | 40º 40' | 00º 42' |
| Lleida | Alcanó | 41º 28' | 00º 37' |
| Teruel | Hinojosa de Jarque | 40º 40' | 00º 46' |
| Teruel | Mezquita de Jarque | 40º 43' | 00º 52' |
