# Supplementary material for: Seedling emergence response of rare arable plants to soil tillage varies by species
Source: PLoS One. 2018 Jun 25;13(6):e0199425. doi: 10.1371/journal.pone.0199425 (PMC6016903; doi:10.1371/journal.pone.0199425)
Supplement: S2 Table — Total annual cumulative emergence the second year (% ± SE) of 30 rare arable plants from seeds sown in till and non-till plots, the tillage treatment; trial repeated in two consecutive seasons (S1, 2013/14; S2, 2014/15), the season treatment. Annual soil disturbance was performed in early autumn in tilled plots sown. The 2nd and 4th columns represent significance for main effects and interactions on emergence analysed by GLM. Values followed by the letter a were significantly higher after analysing each season separately for tillage treatment due to significant interactions. (DOCX) [file pone.0199425.s003.docx]

|  |  | |  |  | **Total Cumulative Emergence** | | | | | | | | | | | | | | | | | | | | |
| --- | --- | --- | --- | --- | --- | --- | --- | --- | --- | --- | --- | --- | --- | --- | --- | --- | --- | --- | --- | --- | --- | --- | --- | --- | --- |
|  |  | |  |  | **No-till** | | | | |  | | **Tilled** | | | | | | | | | | | | |  |
| **Species** | **Tillage** | **Season** | | **Tillage***  **Season** | | **2013/14** | **2014/15** | | |  | | | | | **2013/14** | | | | **2014/15** | | | |  |  |  |
| *Adonis aestivalis* | NS | ** | | ** | | 12.2 ± 2.5 | 41.6 ± 10.0a | | |  | | | | | 19.4 ± 4.9 | | | | 18.9 ± 4.4 | | | |  |  |  |
| *Adonis flammea* | NS | *** | | NS | | 4.7 ± 1.4 | 16.2 ± 1.3 | | |  | | | | | 6.4 ± 1.9 | | | | 13.2 ± 4.9 | | | |  |  |  |
| *Agrostemma githago* | NS | NS | | NS | | 0.3 ± 0.1 | 0.4 ± 0.2 | |  | | | | | 0.2 ± 0.2 | | | | 0.3 ± 0.2 | | | |  |  |  |  |
| *Androsace maxima* | NS | NS | | NS | | 2.8 ± 0.9 | 5.1 ± 2.1 |  | | | | | 3.2 ± 1.5 | | | | 3.6 ± 1.1 | | | |  |  |  |  |  |
| *Asperula arvensis* | NS | *** | | * | | 0.8 ± 0.4 | 15.7± 3.6 | | |  | | | | | 3.1 ± 0.2a | | | | 7.4 ± 3.3 | | | |  |  |  |
| *Bifora radians* | * | NS | | NS | | 0.9 ± 0.6 | 2.0 ± 1.8 | |  | | | | | 2.8 ± 1.1 | | | | 5.2 ± 0.4 | | | |  |  |  |  |
| *Biscutella auriculata* | NS | NS | | NS | | 0.4 ± 0.4 | 1.4 ± 0.8 |  | | | | | 0.5 ± 0.4 | | | | 1.8 ± 0.8 | | | |  |  |  |  |  |
| *Bupleurum rotundifolium* | NS | * | | NS | | 4.1 ± 1.2 | 9.0 ± 4.5 | | |  | | | | | 6.4 ± 0.5 | | | | 11.1 ± 3.2 | | | |  |  |  |
| *Camelina microcarpa* | NS | NS | | NS | | 2.2 ± 0.6 | 2.9 ± 1.2 | |  | | | | | 2.5 ± 0.8 | | | | 5.7 ± 1.5 | | | |  |  |  |  |
| *Cerastium perfoliatum* | * | NS | | NS | | 3.1 ± 1.4 | 4.9 ± 2.6 |  | | | | | 3.5 ± 0.5 | | | | 10.6 ± 2.4 | | | |  |  |  |  |  |
| *Conringia orientalis* | ** | *** | | NS | | 0.9 ± 0.4 | 3.1 ± 0.6 | | | |  | | | | | 2.2 ± 0.8 | | | | 5.3 ± 0.7 | | | |  |  |
| *Consolida orientalis* | NS | ** | | NS | | 8.1 ± 3.0 | 3.2 ± 1.4 | | |  | | | | | 7.2 ± 1.5 | | | | 4.2 ± 1.0 | | | |  |  |  |
| *Consolida pubescens* | NS | NS | | ** | | 2.2 ± 2.1 | 13.4 ± 2.4 | |  | | | | | 9.9 ± 3.0a | | | | 7.3 ± 2.1 | | | |  |  |  |  |
| *Delphinium gracile* | NS | * | | NS | | 2.2 ± 1.8 | 1.1 ± 0.6 | | |  | | | | | 16.5 ± 14.3 | | | | 1.4 ± 0.8 | | | |  |  |  |
| *Delphinium halteratum* | NS | NS | | * | | 8.5 ± 3.0 | 4.3 ± 2.3 | |  | | | | | 5.8 ± 0.4 | | | | 10.3 ± 2.4a | | | |  |  |  |  |
| *Galeopsis ladanum* | NS | NS | | NS | | 2.0 ± 0.7 | 1.3 ± 1.3 | |  | | | | | 1.5 ± 0.4 | | | | 0.6 ± 0.4 | | | |  |  |  |  |
| *Hypecoum pendulum* | * | *** | | NS | | 6.5 ± 2.9 | 1.0 ± 0.5 | | |  | | | | | 13.1 ± 3.7 | | | | 1.5 ± 0.9 | | | |  |  |  |
| *Iberis amara* | NS | NS | | NS | | 3.1 ± 0.9 | 1.8 ± 0.9 | |  | | | | | 3.9 ± 1.2 | | | | 4.0 ± 1.4 | | | |  |  |  |  |
| *Lathyrus aphaca* | NS | *** | | NS | | 0.0 ± 0.0 | 52.7 ± 14.0 | | |  | | | | | 0.0 ± 0.0 | | | | 45.5 ± 4.8 | | | |  |  |  |
| *Legousia hybrida* | NS | *** | | NS | | 1.0 ± 0.4 | 32.0 ± 12.5 | | |  | | | | | 4.0 ± 1.2 | | | | 30.6 ± 8.4 | | | |  |  |  |
| *Neslia paniculata* | NS | ** | | NS | | 2.3 ± 1.1 | 8.0 ± 3.2 | | |  | | | | | 2.6 ± 0.9 | | | | 6.7 ± 1.8 | | | |  |  |  |
| *Nigella gallica* | NS | * | | ** | | 9.2 ± 0.9 | 2.6 ± 1.2 | | |  | | | | | 5.7 ± 2.0 | | | | 6.7 ± 1.2a | | | |  |  |  |
| *Papaver argemone* | * | NS | | NS | | 2.4 ± 1.0 | 0.9 ± 0.4 | |  | | | | | 3.5 ± 0.5 | | | | 4.5 ± 2.2 | | | |  |  |  |  |
| *Papaver dubium* | ** | *** | | ** | | 2.5 ± 0.5a | 0.2 ± 0.1 | | |  | | | | | 0.4 ± 0.3 | | | | 0.4 ± 0.3 | | | |  |  |  |
| *Ranunculus arvensis* | ** | NS | | NS | | 6.2 ± 2.4 | 8.5 ± 2.6 | |  | | | | | 13.6 ± 4.4 | | | | 20.8 ± 2.5 | | | |  |  |  |  |
| *Roemeria hybrida* | NS | *** | | *** | | 10.4 ± 1.1a | 1.0 ± 0.2 | | |  | | | | | 6.3 ± 1.1 | | | | 4.1 ± 1.5a | | | |  |  |  |
| *Silene conoidea* | * | ** | | NS | | 1.1 ± 0.7 | 2.0 ± 0.7 | | | |  | | | | | 1.4 ± 0.3 | | | | 6.0 ± 2.8 | | | |  |  |
| *Thlaspi arvense* | *** | ** | | * | | 1.6 ± 0.5 | 1.0 ± 0.3 | | | |  | | | | | 2.5 ± 0.8 | | | | 8.2 ± 2.6a | | | |  |  |
| *Turgenia latifolia* | * | NS | | NS | | 0.4 ± 0.2 | 0.5 ± 0.2 |  | | | | | 1.0 ± 0.3 | | | | 0.8 ± 0.4 | | | |  |  |  |  |  |
| *Vaccaria hispanica* | ** | *** | | NS | | 2.4 ± 0.4 | 0.2 ± 0.1 | | |  | | | | | 5.1 ± 2.1 | | | | 1.2 ± 0.7 | | | |  |  |  |

***<0.001, ** 0.001-0.01, * 0.01-0.05, NS: not significant.
